# Supplementary material for: Seminoma and Embryonal Carcinoma Footprints Identified by Analysis of Integrated Genome-Wide Epigenetic and Expression Profiles of Germ Cell Cancer Cell Lines
Source: PLoS One. 2014 Jun 2;9(6):e98330. doi: 10.1371/journal.pone.0098330 (PMC4041891; doi:10.1371/journal.pone.0098330)
Supplement: File S1 — ZIP file containing DMRforPairs output for significant regions. Please start from the html files. (ZIP) [file pone.0098330.s008.zip › figures/22549.pdf]

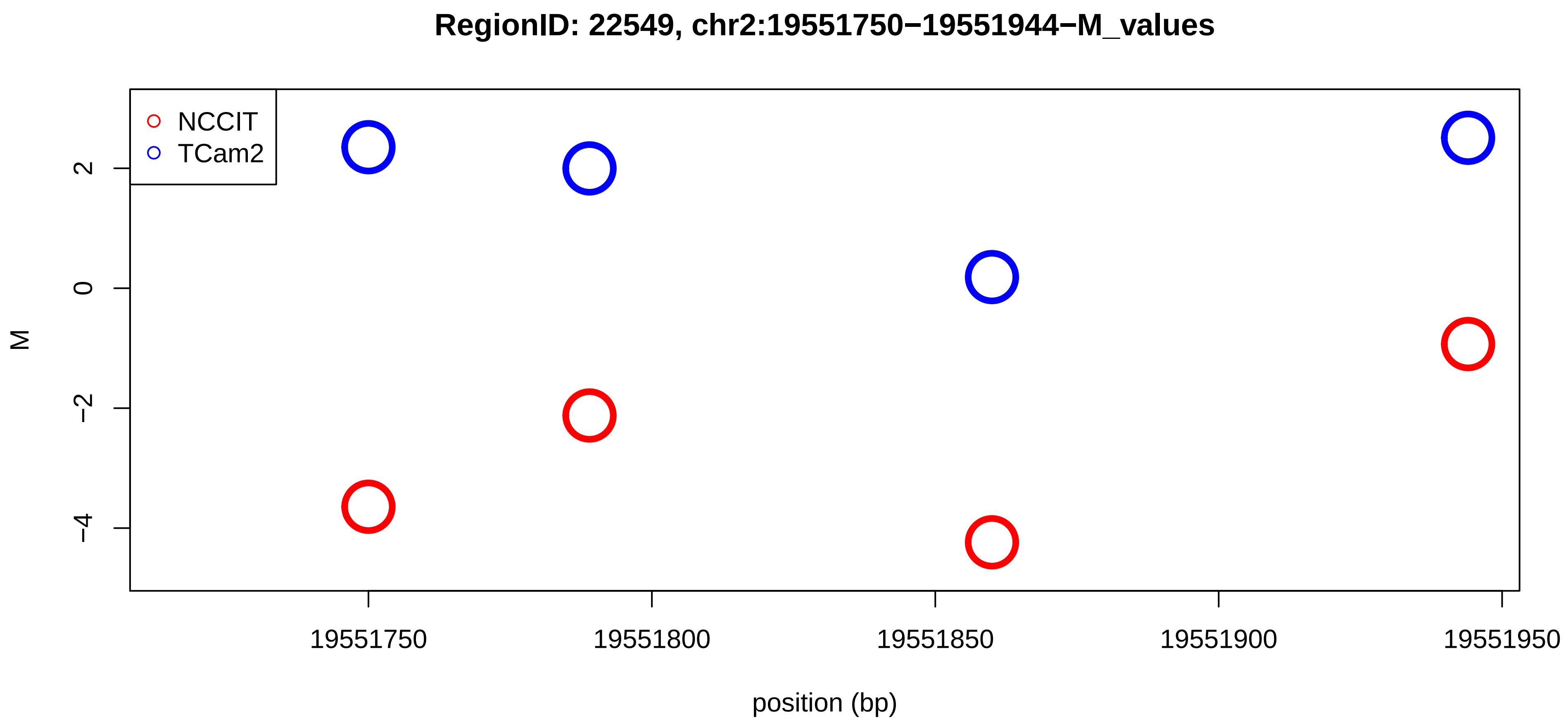
RegionID: 22549, chr2:19551750−19551944−M_values
NCCIT
TCam2
M
2
0
−2
−4
19551750
19551800
19551850
19551900
19551950
position (bp)

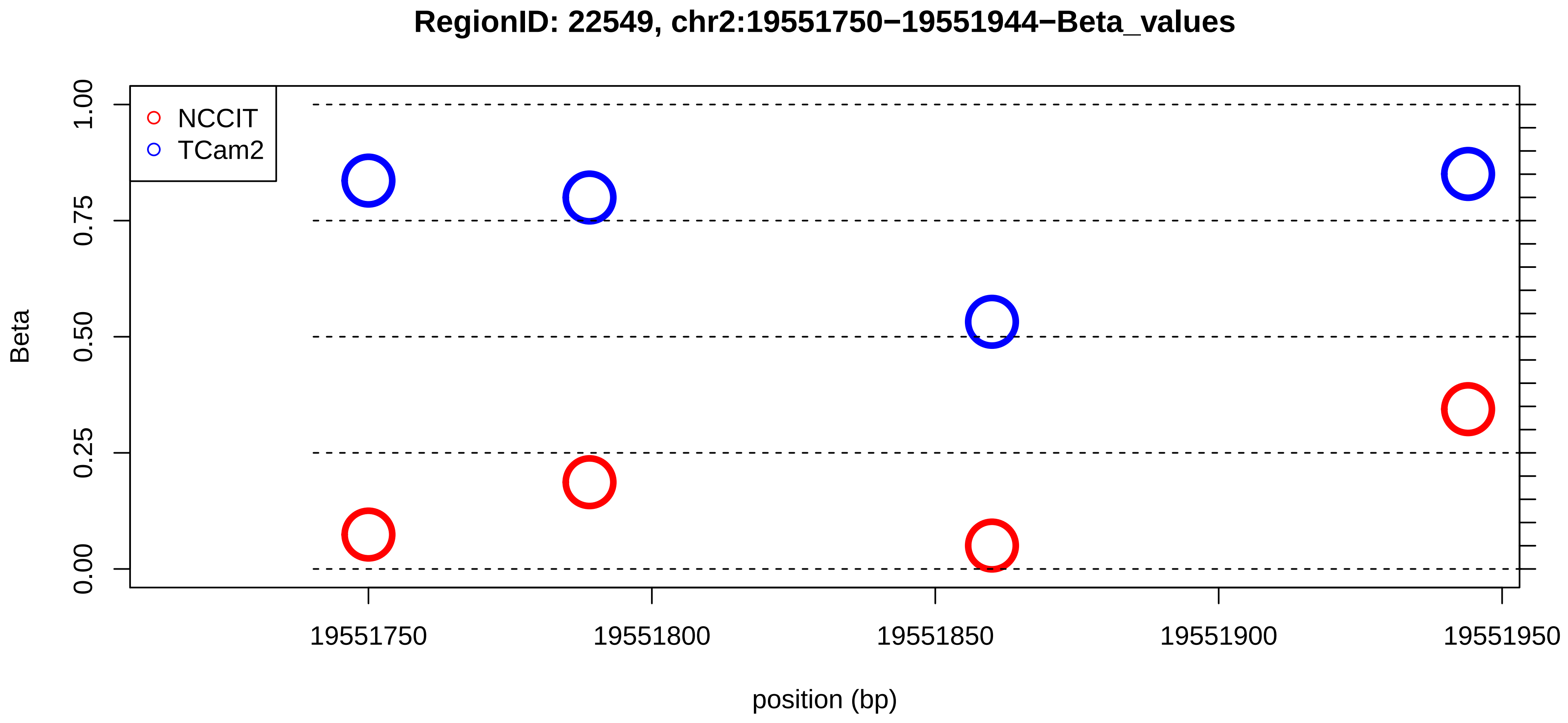
RegionID: 22549, chr2:19551750−19551944−Beta_values
NCCIT
TCam2
Beta
1.00
0.75
0.50
0.25
0.00
19551750
19551800
19551850
19551900
19551950
position (bp)
